# Light's twist

**DOI:** 10.1098/rspa.2014.0633

**Published:** 2014-12-08

**Authors:** Miles Padgett

**Affiliations:** School of Physics and Astronomy, University of Glasgow, Glasgow G12 8QQ, UK

**Keywords:** orbital angular momentum, optical vortex, structured light

## Abstract

That light travels in straight lines is a statement of the obvious. However, the energy and momentum flow within light beams can twist to form vortices such as eddies in a stream. These twists carry angular momentum, which can make microscopic objects spin, be used to encode extra information in communication systems, enable the design of novel imaging systems and allow new tests of quantum mechanics.

In the 1600s, Kepler guessed that the momentum of sunlight caused the tail of a comet to always point away from the sun. However, the corresponding forces are very small, equal to the optical power divided by the speed of light, and hence go unnoticed in our everyday lives. In the early 1900s, by drawing an analogy between light and mechanical systems, Poynting [1] deduced that circularly polarized light carries an angular momentum. We now see this deduction as compatible with our understanding of the ±ℏ spin angular momentum (SAM) of the photon.

A SAM of ±ℏ per photon is consistent with a photon description of the absorption and emission of light from dipole transitions within atomic systems, where the angular momentum is conserved between the electronic state and the interacting optical field. In the 1930s, Darwin (grandson of the originator of evolution) [2] recognized that more complicated transitions required an angular momentum exchange between light and atom corresponding to integer multiples of ℏ. He postulated that this extra angular momentum could be generated as a recoil torque, arising when the centre of mass of the atomic system was slightly displaced from the optical emission axis, and hence that the linear momentum of the light acted on a radius vector. This recoil torque is similar to that created by a garden water sprinkler, which is set spinning in reaction to the jets of water that appear to spiral upwards, even though each water droplet moves in a straight line.

Despite the work of Darwin and others, it was not until 1992 that Allen and co-workers [3] recognized that a light beam possessing helical phasefronts carried an orbital angular momentum (OAM) distinct from, and additional to, the SAM of the photon. They derived that beams with ℓ interwined helical phasefronts carry an OAM of ℓℏ per photon (figure 1). When such a light beam is represented by optical rays, these rays are skewed with respect to the beam direction (much like the jets of water from the above-mentioned water sprinkler) [4]. The helical phasefronts that give rise to OAM mean that the very centre of the beam has an ill-defined phase (in much the same way that the time of day at the North Pole is ill-defined). At this central phase singularity, every phase value has a counterpart that is shifted by *π* radians, resulting in destructive interference and zero on-axis intensity. Therefore, all beams with helical phasefronts have an annular intensity cross section and are sometimes referred to as ‘doughnut beams’.

**Figure 1.**
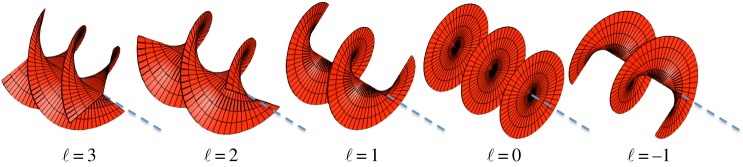
When laser beams have helical phasefronts, their energy and momentum twist around the beam axis, and the beam carries an orbital angular momentum of ℓℏ per photon.

Prior to the recognition of their momentum properties, helically phased beams had been created by Soskin and co-workers [5] as the diffracted orders from gratings modified to have a forked line structure at their centre (figure 2). Such forked patterns are easily formed by spatial light modulators, which use a similar technology to that found in a modern digital projector. This technology forms the basis of much of the past and present research in this area of structured light. Rather than use the spatial light modulator to transform the output of a conventional laser from a Gaussian beam into one with helical phasefronts, recent advances have used the spatial light modulator as part of the laser cavity to control the shape of the output beam itself [6]. Many other methods for generating OAM beams have also been developed, ranging from the use of cylindrical lenses to convert between modal types [7] to helical (spiral) phase plates [8] and liquid crystal waveplates with a spatially varying orientation of their optic axis: so-called Q-plates [9].

**Figure 2.**
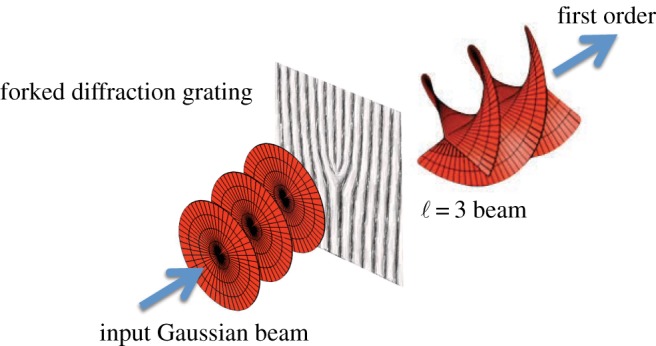
When a standard laser beam illuminates a forked diffraction grating, the first-order diffracted beam has helical phase fronts and consequentially carries orbital angular momentum.

Shortly following the recognition of OAM within light beams was the demonstration, by Rubinsztein-Dunlop and co-workers [10], of the transfer of this angular momentum to macroscopic objects. Their work and subsequent studies were performed within optical tweezers, which conventionally use tightly focused laser beams to trap and then manipulate micrometre-sized dielectric (i.e. transparent) particles [11]. Many groups worldwide have since replaced the Gaussian laser beam in optical tweezers with a tightly focused OAM beam to both trap micrometre-sized particles and set them into rotation by angular momentum transfer [12]. Our own work concentrated on the simultaneous transfer of both SAM and OAM to the same particle, where their relative handedness could give addition or subtraction, to speed up or slow down the rotation rate [13]. For beams that are large compared with the particle, the behaviour of SAM and OAM is different. Whereas the transfer of SAM causes particles to spin around their own axis, the transfer of OAM causes them to orbit around the beam axis, confined at the radius of the doughnut intensity profile, rather like cyclists in a velodrome [14,15] (figure 3). These demonstrations of ‘optical spanners’ raised interest in OAM, with the instant appeal being to create optically driven micromachines [16] or pumps [17]. Spatial light modulators have also been key to the development of this area, initially used by Grier and co-workers [18,19] to create multiple beams, the phasefronts of which can be shaped to give OAM and/or other beam properties—so-called holographic optical tweezers.

**Figure 3.**
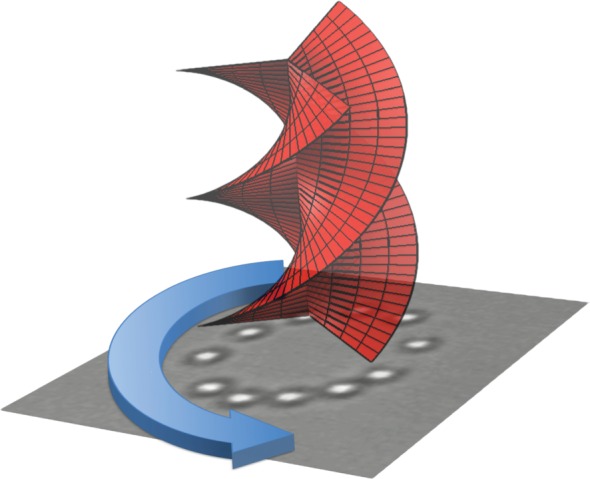
When a helically phased laser beam (ℓ=3) is incident on microscopic particles, they are set into rotation around the beam axis.

Although the spin and orbital components contribute to the total angular momentum of the light, in general they are both generated and interact with the micro- or macroscopic world in different ways. The SAM is manifest in the polarization state of the light. The importance of the polarization state is well understood, and key optical components for its manipulation include birefringent waveplates that introduce a phase delay between orthogonal linear states and optical activity that introduces phase delay between circular states. For OAM, the mathematically analogous states to circular and linear polarization are the helically phased Laguerre–Gaussian laser modes and the Hermite–Gaussian modes, respectively. A birefringent waveplate has no effect on the mode, and hence OAM, of the transmitted light, but a modal transformation can be introduced by clever use of cylindrical lenses [7] or the inversion properties of a Dove prism. Similarly, optical activity has no bearing on the OAM state of the light [20]; rather, the mathematically equivalent transformation is one of image rotation [21]. Sometimes, one encounters physical processes that act equivalently on both the SAM and OAM, e.g. total absorption of the light by a macroscopic object. More subtle equivalent processes include a rotation of the reference frame, where a beam carrying both SAM and OAM experiences a phase delay proportional to the total (*spin*+*orbit*) angular momentum [22]. Another process is the action of a spinning window that introduces photon drag [23] rotating both the polarization and image formed by transmitted light through the same (small) angle [24,25]. However, in general, the SAM and OAM are distinct phenomena and are not interchangeable. Nowhere is this difference more apparent than in the interaction of individual atoms with the light beam: whereas the SAM is associated with the selection rules of Zeeman transitions, the OAM mainly introduces azimuthal components to the recoil shift of the atom [26]. Fundamentally, the decomposition of an OAM-carrying beam into its various OAM components depends upon the choice of measurement axis [27], whereas a description of its polarization state does not.

Another early study of beams carrying OAM centred around nonlinear optics. When an intense infrared laser is focused into a range of exotic man-made crystals, the nonlinearity of the polarizability of the crystal means that a significant fraction of the optical energy can be re-emitted at twice the optical frequency of the incident light. This frequency doubling actually forms the basis for many modern laser systems, which are inherently infrared in the lasing transition but, thanks to the inclusion of a nonlinear crystal, emit visible light. As described by a simplistic photon picture, the energies of two infrared photons from the input laser are combined to form one green photon of twice the energy and hence twice the optical frequency (figure 4). In addition to having twice the energy, it transpires that the output photon also carries the sum of the OAM of the input photons [28]. This conservation of OAM within the optical fields is of vital importance for generation of entangled photon pairs in the study of OAM's quantum properties.

**Figure 4.**
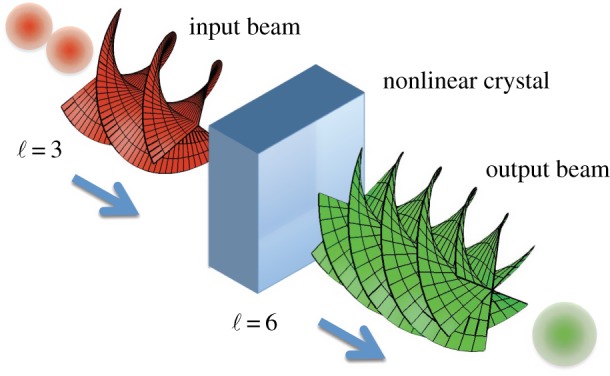
Nonlinear crystals can double the frequency of an incident laser beam, conserving the energy in the optical fields. In addition to the conservation of energy, the conservation of momentum leads to a doubling of the OAM per photon.

Helically phased beams carrying OAM also have uses in optical imaging systems. Just as a forked diffraction grating can transform a Gaussian laser beam into a helically phased beam, when incorporated into an imaging system, the grating transforms the point spread function of the system into an annular ring. An early recognition of this doughnut-shaped point spread function led to proposals to incorporate similar optical elements into a telescope. This incorporation transforms the image of a bright star into an annular ring, enabling the observation of nearby objects such as much fainter stars or potentially orbiting planets [29]. A similar approach can be applied to microscopy, where the inclusion of an OAM transforming element also gives an annular point spread function and a new kind of phase-contrast imaging modality leading to omnidirectional edge enhancement of phase objects [30]. If combined into an interferometer, the helically phased reference wave means that the interference fringes produced by a surface feature are spirals, the handedness of which distinguishes up from down, breaking the ambiguity of conventionally produced concentric fringes [31].

Returning to more basic optical properties, whereas the SAM per photon can only take one of two values, the OAM can take any integer value of ℓ. Taking each integer state to represent a letter of the alphabet within a communication system, one sees immediately that OAM may enable systems with additional information capacity. We made an early demonstration of an optical communication system using OAM to create eight distinct spatial states within a single optical beam [32], an approach which is now being adopted by many research groups in their quest to raise the capacity limit of free-space communication systems [33]. Although free-space propagation is perhaps the simplest and most obvious configuration, albeit one which is subjected to atmospheric aberrations [34], research is also proceeding in the use of OAM within specially designed multi-mode fibres [35].

Key to taking advantage of the multi-valued nature of OAM are the technologies by which the OAM can be generated and measured. Measuring polarization is easy—a polarizing beam splitter separates any incoming beam into its two states. What is needed for OAM is a beam splitter with many different outputs, so that each output can be mapped to a particular OAM value. Separating multiple optical states is not new: a lens focuses to a position dependent upon the direction, i.e. lateral momentum, of the incoming light. A similar idea can also work for OAM. Using bespoke optical components, we showed that the doughnut of an OAM beam can be unwrapped into a straight line, mapping the annular phase into a lateral variation, and therefore allowing the OAM states to be separated using a simple lens [36]. Such a device seems to be an essential technology if OAM is ever to find widespread use as a carrier of information [37].

This increased alphabet also has uses in studies and applications of quantum science [38]. The spooky behaviour of quantum mechanics is present in the correlations that exist between particles even when they are arbitrarily distant from each other. Most of the early demonstrations of quantum entanglement used the SAM of the light, manifested as polarization, to perform experimental tests in support of quantum theory. Zeilinger and co-workers [39] used OAM to extend these tests to cover multi-valued systems. As discussed previously, frequency doubling within a nonlinear crystal conserves OAM. However, nonlinear crystals can also be used in reverse to convert a single incident photon into two photons of lower frequency in a process called ‘parametric down-conversion’. The OAM is again conserved, although there is no requirement for it to be split equally between the photons produced. The same forked diffraction gratings used to generate OAM can be used to measure the OAM by converting the helically phased beam into a Gaussian beam that can then, and only then, be coupled into a single-mode fibre for subsequent detection using a conventional single-photon detector. Typically, the photons that are incident upon the nonlinear crystal have no OAM, whereas the OAM of the two photons produced is constrained by the conservation of OAM to be of equal magnitude but opposite sign, even when the OAM value is very high [40]. This conservation applies not just to the measurement of single states, but also to fractional OAM states [41] and superpositions, where both magnitudes and phases between the two separated measurements are correlated [42]: the hallmark of quantum entanglement.

Our own work established an angular form of the Heisenberg uncertainty principle relating the uncertainty in OAM to an uncertainty in angular position [43]. In the well-known uncertainty principle, the precision of a particle's position is related to the precision of its linear momentum through the product of their standard deviations as $\Delta x\times \Delta p_{x}\geq \hbar /2$. For angular momentum, the complementary variable is angular position. However, unlike linear position, angular position is bounded to lie within a range ±*π*, which leads to a more complicated form of uncertainty relationship. Despite this complication, for small uncertainties in angular position (Δ*θ*), an angular uncertainty relationship can be written as Δθ×ℏΔℓ≥ℏ/2. Within quantum physics, the Heisenberg uncertainty relationship leads naturally to the Einstein–Podolsky–Rosen quantum paradox, which our own work using OAM has extended to cover the angular case [44], establishing the feasibility of using OAM within quantum studies and quantum applications.

OAM-carrying beams and their inherent annular intensity profile are an excellent choice for creating traps for cold atoms. When the light is blue detuned from the transition frequency, the atoms experience an optical dipole force away from the high light intensity, meaning that they can be trapped in a region of near darkness at the centre of the beam(s) where they are not subjected to unwanted heating from residual scattering of the optical field [45], allowing extremely high atomic densities to be achieved [46].

Even within classical physics, OAM has led to new insights of optical effects. For example, the Doppler shift is a well-known effect used to measure the velocity (*v*) of approaching objects (e.g. radar speed guns). We have recently applied the Doppler effect to OAM, measuring the rotational speed of macroscopic objects even when the linear Doppler shift is zero [47], and similar work has been reported as applied to particles [48,49].

The physics behind the Doppler shift can be expressed in a number of equivalent formulations. One derivation of the linear Doppler shift is based on the recognition that a light beam exerts a force on an approaching object equal to the power in the light beam (*P*) divided by the speed of light. The movement of the approaching object does work (WD) against this force, transferring energy to the light beam, *WD*=(*P*/*c*)×*v*. Given that the energy per photon is ℏω, the number of photons incident per second is N=P/ℏω. Equating the work done per photon to the resulting shift in the photon's frequency, we obtain ℏΔω=(P/Nc)×v and hence the usual expression for the linear Doppler shift of Δ*ω*=(*v*/*c*)×*ω*. However, rather than doing work against a linear force, it is also possible to do work against the torque exerted by the light (figure 5). For a light beam carrying OAM of ℓℏ per photon, the torque exerted on a scattering object is given simply as ℏΔℓ. Assuming that the scattered light is spatially incoherent and hence (on average) carries no angular momentum, then the work done per photon on the light by the rotation (*Ω*) of a scattering surface is ℏΔω=ℏΔℓ×Ω, giving a rotational Doppler shift of Δ*ω*=ℓ*Ω* [50,51]. This rotational frequency shift can be non-zero even when the linear velocity, and hence the linear shift, is zero. Note also that, unlike the linear shift, this rotational shift is independent of the optical frequency, and hence each spectral component of the scattered light is shifted by the same value. Consequently, when scattered from a rotating surface, even a white-light OAM source gives rise to a single-valued frequency shift [52].

**Figure 5.**
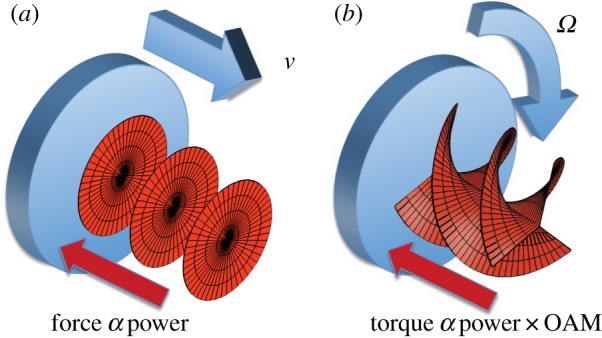
The linear Doppler shift can be derived from the work done against the force exerted by an incident light beam (*a*). Similarly, a rotational Doppler shift can be derived from the work done against a torque (*b*).

At first sight, the OAM of light seems to require specialist equipment to be created within an optics laboratory, but, in fact, phase singularities and the surrounding optical vortices are a generic occurrence within all light fields. Whenever three or more plane waves interfere, the resulting interference pattern contains a network of phase singularities, positions of perfect darkness around which the phase advances or retards by 2*π* [53,54]. Perhaps the best-known example of such a pattern is the optical speckle observed whenever a laser beam is scattered from a rough surface. Rather than being of a uniform intensity, the scattered light appears to contain numerous specks of darkness, all of which mark phase singularities, a phenomenon observed and originally discussed by Nye & Berry [55] for the case of scattered ultrasound. However, the interference that gives rise to these singularities does not just occur in one plane, but rather throughout the volume that the waves interfere. Therefore, in three dimensions, rather than being points, these phase singularities describe dark lines embedded in the light. For the specially prepared OAM-carrying beams, the dark lines are straight and lie along the beam axis, but for the more general case of optical speckle, we showed that the lines are described by Brownian random walks that either percolate through space or form closed loops [56]. Occasionally, or by specific design, these loops can form topological features such as links or even more elaborate knots [57]. In day-to-day life, this topological nature of light's darkness is hard to observe but is present within each spectral component of any scattered light.

As noted above, the early studies of phase singularities were not optical but acoustic [55]. This highlights that OAM is not solely an optical phenomenon, but a potential property of any wave-like system. Indeed, OAM has been specifically explored in acoustics [58,59], electron beams [60,61] and in the extremes of the electromagnetic spectrum [62,63].

Over the past 20 years, OAM has attracted significant interest in applications ranging from optical manipulation to imaging. Beyond applications, OAM has provided new insights into problems ranging from rotational frequency shifts to angular uncertainty, and, in quantum science, OAM gives a basis set for new tests and demonstrations of high-dimensional entanglement. Most generally, OAM has reminded us that intensity alone is not enough and that the phase structure of the light beam is of equal importance.

It remains an open question whether OAM will have a lasting impact upon commercially significant applications. Perhaps the most promising of these applications is free-space communications, where OAM offers an approach to spatial division multiplexing. However, the number of low-loss channels an optical system can support is dependent upon its Fresnel number. OAM is just one choice of a possible set of states, and whether it is the best choice for communication depends upon the emergence of key technologies around switching between, and sorting, OAM states (a work in progress). Another possible application area for OAM is within imaging systems, where astronomy, interferometry, omnidirectional edge-enhancement and even stimulated emission depletion microscopy may all benefit from OAM-carrying beams. Fabricating OAM filters that combine optical efficiency with modal purity and programmability will be key to incorporating OAM into such imaging systems. Finally, the multi-state nature of OAM might provide a defining tool within quantum information processing, an opportunity that can only be made easier by improved low-loss componentry and schemes for logic operations and logic gates.

In the 20 years since OAM was articulated by Allen *et al*. [3], a vibrant and supportive community has been established. Hopefully, this community will continue to use OAM to both inform new fundamental optics and enable new applications.

## Author profile

**Miles Padgett** holds the Kelvin Chair of Natural Philosophy at the University of Glasgow and is a Royal Society/Wolfson Merit Award holder. In 2001, he was elected to fellowship of the Royal Society of Edinburgh, of the OSA in 2011, of SPIE in 2012 and in 2014 a Fellow of the Royal Society—the UK's National Academy. In 2008, he won the Institute of Physics Optics and Photonics Prize; in 2009, the Institute's Young Medal; and in 2014, the Kelvin Medal of the Royal Society of Edinburgh. He is internationally recognized for his leadership in the field of optics and in particular of optical momentum. His best-known contributions include an optical spanner for spinning micrometre-sized objects, use of orbital angular momentum to increase the data capacity of communication systems and an angular form of the Einstein–Podolsky–Rosen quantum paradox.
